# Concentration of Rutin Model Solutions from Their Mixtures with Glucose Using Ultrafiltration

**DOI:** 10.3390/ijms11020672

**Published:** 2010-02-09

**Authors:** Swallow Wei, Md. M. Hossain, Zaid S. Saleh

**Affiliations:** 1 Department of Chemical & Materials Engineering, The University of Auckland, Private Bag 92019, Auckland, New Zealand; 2 Department of Chemical & Petroleum Engineering, UAE University, P.O. Box 17555, Al Ain, United Arab Emirates; 3 The New Zealand Institute for Plant & Food Research Limited, Private Bag 92169, Auckland, New Zealand

**Keywords:** polyphenols, rutin, sugar, ultrafiltration, fouling, concentration factor

## Abstract

Separation of polyphenolic phytochemical compounds from their mixtures with sugars is necessary to produce an added-value sugar-reduced extract with high biological activity from fruit juice processing industry waste streams. The separation characteristics of a binary mixture of rutin and glucose using a Pellicon-2 regenerated cellulose ultrafiltration membrane with an area of 0.1 m^2^ having nominal MWCO of 1,000 Da were investigated, to demonstrate the separation of phenolic compounds from sugars. The effects of the operating variables–transmembrane pressure, feed solution temperature and pH, initial feed concentration and feed flow rate–on the permeate flux and enrichment of rutin, were determined. The permeate flux increased with the increase in transmembrane pressure up to a certain limit and after that the flux remained more or less constant. The optimum transmembrane pressure was within 4–5 bar. The flux increased with the increase in feed solution temperature because of reduced feed viscosity, and better solubility. The concentration of rutin was optimum at lower temperature (30°C), with an enrichment factor of 1.3. The effect of pH on permeate flux was less obvious. Lowering the feed solution pH increased the retention of rutin and the optimum separation was obtained within pH 3–4. The permeate flux decreased with the increase in feed concentration of rutin (concentration range 0.1–0.5 g/L). The enrichment of rutin was significant in the glucose concentration range 0.35–0.5 g/L. The feed flow rate had a significant effect on the flux and separation characteristics. Higher cross-flow through the membrane reduced the fouling by providing a shear force to sweep away deposited materials from the membrane surface. At high feed flow rate, more rutin was retained by the membrane with less sugar permeating through. The optimum feed flow rate was 1.5 L/min. For the separation of rutin (in the retentate) and glucose (in the permeate), the best results were obtained at rutin enrichment of 2.9 and recovery 72.5%, respectively. The performance of this system was further improved by operating it in a diafiltration mode, in which only approx. 11% of glucose remained in the retentate.

## 1. Introduction

Polyphenols are the most plentiful secondary metabolites in plants [1]. Including their functional derivatives there are more than 8,000 currently known phenolic structures, which are all characterized by an aromatic ring bearing one or more hydroxyl groups [2].

Fruits such as apple possess a wide diversity of polyphenolic compounds, which can be classified into five major groups: flavan-3-ols, hydroxycinnamic acids, dihydrochalcones, anthocyanins and flavonoids [3]. Polyphenols in apple juice are responsible for flavor, color, bitterness and astringency [4]. These polyphenols can also polymerize with protein to form complexes that cause haze and browning during storage of clear apple juice [5].

Polyphenols provide many health-related functional benefits. They can help cellular processes in the human body by their natural antioxidant properties, prevent oxidation of high-density lipids (HDL) and remove low-density lipids (LDL). In addition, because of their ability to absorb free radicals, they can reduce ulceration and negate the effects of mutagens, including carcinogens [1].

Consumer desire to improve health through food and beverage consumption is of vital importance to the fruit and vegetable industries. The various waste streams from apple juice production processes are good sources of polyphenolic phytochemicals [6], which potentially can be added into functional foods and beverages to enhance their health properties [7].

Conventional extraction methods for polyphenols involve organic solvents, such as methanol, ethanol and hexane. Although these solvents are quite effective, the extracts may contain residual solvents that are toxic and unsafe for human consumption [1]. Another method currently being used for large-scale extraction is supercritical fluid extraction (SFE), where polyphenol extraction is performed under high pressure. This method is food safe and efficient, but is expensive to set up and the high temperatures required to increase the extraction rate may degrade the polyphenols [8].

Typically, apple polyphenols are recovered from pomace and pectin extracts, which are readily available, inexpensive and usually characterized by high polyphenolic content [9]. The crude extracts are commonly purified and concentrated by applying, for example, ultrafiltration or resin adsorption. However, these advanced technologies are often used without systematically optimizing the parameters.

Resin adsorption has proven to be an efficient technique for the selective enrichment and recovery of polyphenolic plant secondary metabolites. Adsorption using styrene-divinylbenzene or acrylic polymers is currently performed on an industrial scale, for example to de-bitter citrus products [10,11], standardize and stabilize juices and juice concentrates, and remove browning reaction products or compounds that may precipitate during storage [12,13]. Recent studies have shown that adsorbent resins may also be used for the recovery of valuable compounds from citrus juices and by-products thereof [14–17]. Adsorption technology can be easily integrated into existing production lines and allows semi-continuous operation [9]. Although widely applied in the food industry, there are few studies that systematically evaluate the process parameters to obtain classes of phenolic compounds selectively using polymeric adsorbents, thus rendering the industrial process development mostly empirical [18–22].

Membrane filtration is favored over other conventional extraction methods because toxic solvents can be avoided [1]. The other advantages of membrane filtration, especially ultrafiltration, are easy automation [24] and scaling up [1], shorter processing times [23], lower labor and energy costs [9], less waste to be disposed [24], no additives, and moderate operation conditions [1]. However, the main disadvantage of membrane filtration is membrane fouling, resulting in reduced separation as measured by a decline in permeate flux. This is caused by deposition and accumulation of solute or colloidal particles at the membrane surface or inside the pores [25].

For apple juice, the main issue associated with using membrane filtration is co-fractionation of sugars with polyphenolic compounds, which dominate the composition of the extract [26]. Therefore, research on developing methods for separating sugars and polyphenolics is continuing, in order to produce concentrated polyphenolic fractions that contain minimal amounts of sugar.

The objectives of this research are thus: (1) to evaluate the applicability of membrane ultrafiltration for the concentration and separation of polyphenolics from their mixtures with sugars; (2) to study the effects of operating parameters, such as pH, temperature, initial concentration of the polyphenolics and feed flow rate, on the concentration factors and flux through the membrane and (3) to study the effect of the mode of operation, i.e. diafiltration, on the separation characteristics of the process.

## 2. Experimental

### 2.1. Preparation of Feed Solution

The feed solution was firstly prepared using single sugars or polyphenolic compounds. Rutin (molecular weight 610 Dalton) and chlorogenic acid (molecular weight 340 Dalton), representing the polyphenolic compounds in apple juice, were sourced from Sigma (USA). Glucose (molecular weight 180) and sucrose (molecular weight 360), representing the mono and disaccharides in apple juice, were purchased from Healtheries and Chelsea, respectively. Because of the poor solubility of rutin in deionized water, 5% by volume ethanol (AR grade) was added into the rutin solution, followed by mixing in an ultrasonic bath at 45 °C for 30–60 minutes (depending on the rutin concentration). The binary solutions were made with rutin and glucose. The concentrations of the single-component feed solution were: 0.075 g/L chlorogenic acid, 0.25 g/L rutin, or 0.25 g/L glucose. The two-component feed concentrations were: 0.1 g/L to 0.5 g/L rutin and 30 g/L to 80 g/L glucose to make the concentration comparable to single strength fruit juices. Feed pH was adjusted using solutions of a 5% sodium hydroxide (BDH, UK) or 10% phosphoric acid (Orica-Chemnet, New Zealand).

**Figure 1.**
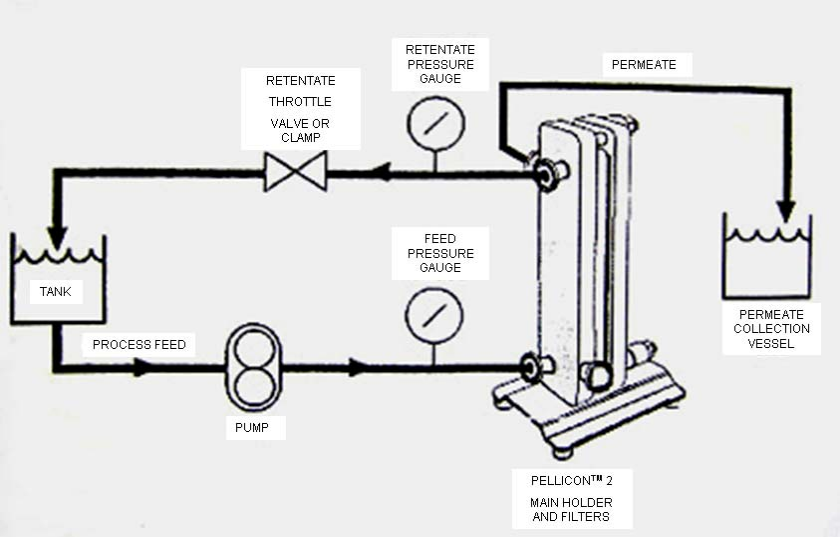
A schematic view of the experimental set-up of ultrafiltration

The experimental rig (Figure 1) consisted of a 10-liter feed tank, immersed in a 20-liter thermostatic hot water bath. The temperature of feed solution was monitored manually using a thermometer. Feed flow rate was controlled by the pump speed controller. Inlet pressure (at the feed side) and outlet pressure (at the retentate side) were monitored by two pressure indicators. Transmembrane pressure was controlled by the valve on the retentate side. The retentate solution from the membrane was recycled into the feed tank, and the permeate solution was collected in a separate bucket. The experiment was performed until a constant ratio of initial feed volume to the final volume was reached. A commercially available membrane module, based on a commercially available Pellicon-2 regenerated cellulose membrane with area of 0.1 m^2^, was obtained from Millipore, MA, USA. It is a hydrophilic membrane with a nominal molecular cut off (MWCO) of 1000 Da. The important characteristics of the membrane are a maximum operating temperature of about 45 °C and the maximum operating pressure is approx. 6 bar. The pump used was a Hydro-cell G-03 series (shaft-driven), with a stainless steel head. The maximum allowable pressure in this pump is 69 bar and maximum flow rate is 11.3 L/min at 1,750 rpm.

### 2.2. Experimental Procedures

This investigation began with ultrafiltration of single components: single sugar and polyphenolic compounds, followed by two-component solution, and finally diafiltration. Before sample filtration, the membrane was flushed by de-ionised water for 60 minutes in order to remove the cleaning solution in the filtration system and storage solution in the membrane. After initial water flushing, pure water flux at room temperature and at 1.5 L/min feed flow rate were measured at different transmembrane pressures (1.5–5 bar) in order to test the membrane permeability and detect fouling. The feed was pumped through the membrane with controlled feed flow rate in the range of 1–2 L/min. The transmembrane pressure was controlled by the retentate valve to give a pressure in the range 2–5 bar. The permeate flow was measured every minute until it was constant. Samples were collected from initial feed solution, final permeate and final feed solution (retentate). Both permeate and retentate samples were also collected at each half hour during filtration process. After each run, the membrane was washed by de-ionised water for 60 minutes and then cleaned by recycling 0.1 M NaOH solution (35 °C, feed flow rate 1.0–1.5 L/min and transmembrane pressure 1 bar for 30–60 minutes). After cleaning, the membrane was stored in 0.1% sodium metabisulphite solution (Orica-Chemnet, New Zealand) in the refrigerator, as recommended by the membrane supplier.

The effects of transmembrane pressure, feed conditions (temperature, pH and concentration) and flow rate were studied to determine the optimum operating conditions. The transmembrane pressures examined were between 2 to 5 bar. The pressure was controlled via the pressure regulator mounted on the retentate section. The temperature effect was studied with temperatures starting from 30 °C to 40 °C. A water bath with a thermostat controller was used to provide constant feed temperature. The effect of pH was studied in the range of 2 to 5, as reported in most studies in the literature. Increasing pH beyond 6 is not recommended because polyphenolic compounds tend to hydrolyse in basic environments. The effects of feed concentration and feed flow rate were also investigated.

### 2.3. Analysis of Membrane Performance

Sugar concentration (°Brix) was measured using a digital refractometer. These °Brix values were converted to g/L using a calibration curve of standard glucose solutions. High Performance Liquid Chromatography (HPLC) was used to analyze polyphenolic concentration. The concentration of polyphenolic compounds was measured by HPLC, using a Phenomenex Synergi 4 μm Hydro RP 80 Å column (250 mm × 4.6 mm). The separation was carried out at 35 °C with a 40 μL injection volume and gradient elution. The binary mobile phase was made up of two solutions: (A) consisting of water/acetonitrile/formic acid 92:5:3 (v/v) and (B) acetonitrile containing 0.1% (v/v) formic acid. The flow rate of separation was 1.5 mL/min in the gradient series as follows: 0–5 min 100–91.3% A, 15–25 min 91–83% A, 25–30 min 83–80% A, 30–39 min 80–70% A, 39–43 min 70–50% A, 43–48 min 50–5% A, 55–65 min 100% A. The concentrations of polyphenolic compounds were determined at 280 nm using standards of known concentration.

The applied pressure, normally called transmembrane pressure (TMP, in pascals), is the average pressure difference between the permeate side and retentate side of the membrane (Equation 1):

(1) $$\text{TMP}=\frac{P_{\text{in}}+P_{\text{out}}}{2}+P_{p}$$

where *P*_in_ and *P*_out_ is the inlet and outlet pressure of the membrane, *P*_p_ is the pressure at the permeate side (considered to be zero in this investigation).

The pure water flux was measured (Equation 2) at a standard transmembrane pressure and temperature, in order to determine the degree of fouling during the separation process. Permeate flux was calculated by taking into account the active surface area of the membrane:

(2) $$J=\frac{1}{A}\frac{dV}{dt}$$

where *J* is permeate flux (Lm^−2^h^−1^), A is membrane area (m^2^), V is permeate volume (L) and t is time (hrs).

The membrane performance was measured by concentration factor and recovery of a certain species. The concentration factor (*C*_*Fi(R,P)*_) is the concentration (C) of species in the retentate (R) divided by its concentration in the initial feed (f) solution (Equation 3):

(3) $$C_{Fi(R,P)}=\frac{C_{i(R,P)}}{C_{i(f)}}$$

Recovery (R, %) of a species was obtained by the mass (kg) in either permeate or retentate divided by its mass (kg) in the initial feed solution (Equation 4):

(4) $$R_{i(R,P)}=\frac{C_{i(R,P)}\times V_{\left(R,P\right)}}{C_{i(f)}\times V_{\left(f\right)}}\times 100\%$$

In order to compare the separation efficiency by recovery (%), the ratio of the volume of retentate to the initial volume of feed was maintained constant (Equation 5). This constant rate is referred to volume concentration rate (VCR):

(5) $$VCR=\frac{V_{F}}{V_{R}}$$

## 3. Results and Discussions

### 3.1. Pure Water Flux

The pure water flux trials were carried out at different operating temperatures (18 °C, 30 °C, 35 °C, and 40 °C) and different feed flow rates (1 L/min, 1.5 L/min). In these trials the transmembrane pressure was the variable being controlled. A linear increase of pure water flux with pressure and temperature was observed, as no fouling had occurred at this stage [28]. On the other hand the feed flow rate had little effect on the pure water flux for a given value of transmembrane pressure, as there would be no concentration polarization on the membrane surface [30]. However, if tap water is used, the flux behaviour will be different, as the tap water contains organic and inorganic compounds which can be easily deposited on membranes and adsorbed into membrane pores [31].

### 3.2. Single Component Ultrafiltration

Initial trials with glucose, sucrose, chlorogenic acid or rutin alone were carried out under constant operational conditions: transmembrane pressure of 4 bar, temperature of 35 °C and feed flow rate of 1 L/min. All the experiments were performed at a constant VCR and over a similar time.

**Figure 2.**
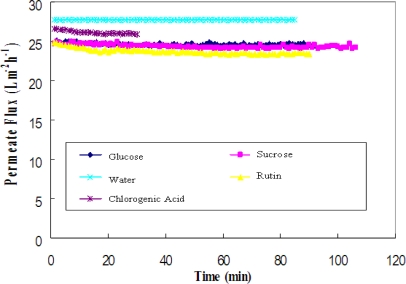
Permeate flux versus time for the solutes (glucose, sucrose, pure water, rutin and chlorogenic acid).

The permeate fluxes of pure water, 0.075 g/L chlorogenic acid, 0.25 g/L glucose, 0.25 g/L sucrose and 0.25 g/L rutin solution are compared in Figure 2. The permeate flux of a 0.25 g/L single-component sugar solution was approximately 90% of the pure water flux. Although the molecular weights of the solutes are different for glucose (180), sucrose (360) and rutin (610), the values of the initial flux were similar because of low feed concentrations. Flux decline for glucose and sucrose was negligible, whereas the larger decline for rutin solution was probably the result of having a larger molecular weight, which is more likely to cause fouling on the membrane. Ultrafiltration of a single-component solution of chlorogenic acid (0.075 g/L) gave a higher permeate flux, which was 95% of the pure water flux.

Table 1 compares the concentration in the retentates of glucose (55 g/L), sucrose (55 g/L), rutin (0.25 g/L) and chlorogenic acid (0.075 g/L). As expected, the solute with the larger molecular weight (rutin) was retained better by the membrane and higher concentration factors were obtained. It was noticed that, although the molecular weight of glucose is much smaller than the membrane MWCO, the applied transmembrane pressure might not have been high enough to overcome the osmotic pressure caused by the glucose concentration gradient across the membrane. As a result, glucose was not concentrated in the permeate solution.

### 3.3. Ultrafiltration of Two-component Solutions

The standard operating conditions were: transmembrane pressure 4 bar, temperature 35 °C, pH 5, feed rutin concentration 0.25 g/L, feed glucose concentration 55 g/L and feed flow rate 1 L/min. The operating conditions, except for the one whose effect was examined, were kept constant at the standard values. Repeats show that the experimental errors were within 5–8% for most of the experiments. Results are summarized in Table 2. The fouling deposits (the last column of the table) were calculated from the overall mass balance of the components in the feed solution (the details are discussed in the mass balance section 3.5). The amounts were calculated per unit area of the membrane and ranged from 0.7 to 4.4 g/m^−2^. These values are only approximate and indicate that the fouling occurred under the aforementioned operating conditions. For more accurate determination of fouling deposits, quantitative measurements using infrared microscopy on the membrane surface would be required.

#### 3.3.1. Effect of transmembrane pressure

**Figure 3.**
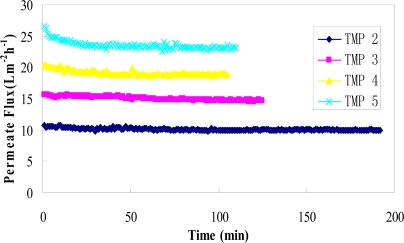
Permeate flux versus time for the binary mixture of rutin and glucose at four transmembrane pressures (TMP = 2, 3, 4 and 5 bar).

At a constant transmembrane pressure, higher fluxes were obtained at the beginning of the ultrafiltration process (Figure 3), followed by a rapid flux decline and finally leveling off. According to Lahoussine-Turcaud *et al*. [32], the flux *vs*. time curve can be divided into two domains: domain 1 corresponds to the initial flux decline for t→0, which may be attributed to internal membrane fouling; domain 2 corresponds to the remaining curve for t >> 0, which may be attributed to external fouling caused by concentration polarization and gel layer formation [23]. During the ultrafiltration process, concentration polarization is often assumed to be constant, whereas the fouling and polarization effects can be superimposed [24].

Figure 3 shows the variation of flux with process time at different transmembrane pressures with a constant temperature of 35 °C, feed flow rate of 1 L/min, feed rutin concentration of 0.25 g/L, feed glucose concentration of 55 g/L (approx. 5 °Brix), and pH of 5. A general trend of increased permeate flux with the increasing transmembrane pressure was observed. At higher TMP of 5 bar, two domains were clearly observed: the initial drop of permeate flux followed by attainment of a constant value. The process reached a steady-state flux after approx. 30–40 min in all runs. After the process reached this state, the flux remained constant over a long time. The flux decline at higher TMP can be explained by the effect of pressure on membrane pore size and gel layer porosity [33]. At high transmembrane pressure, pore size and gel layer porosity decrease, which results in increase of the hydraulic resistance and more rapid flux decline [34].

**Figure 4.**
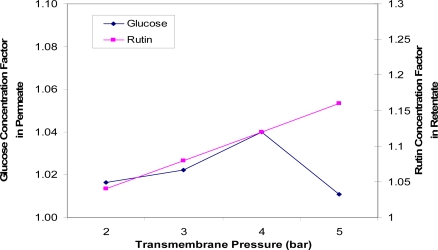
Concentration factors of glucose (in the permeate solution) and rutin (in the retentate solution) versus transmembrane pressures (TMP = 2, 3, 4 and 5 bar).

Figure 4 indicates the effects of transmembrane pressure on the concentrations of glucose and rutin. The optimum separation is a higher glucose concentration in the permeate solution and a higher rutin concentration factor in the retentate, while maintaining permeate flux as high as possible. A rutin concentration factor of 1.12 in the retentate was achieved at 4 bar with good partitioning of glucose in the permeate solution. In general, the higher the transmembrane pressure, the more glucose (in the permeate side) and rutin (in the retentate side) will be transferred through the membrane. However, it was noticed that the glucose concentration factor dropped slightly at 5 bar. This can be explained by high pressure adding additional membrane resistance by compressing the rejected solutes into a thicker or denser fouling layer [9]. The optimum transmembrane pressure was indicated to around 4 bar.

Similar separation and concentration of polyphenolic phytochemical compounds from their mixtures could be obtained when using any clear fruit juice solution such as pear juice, grape juice, boysenberry juice, and blackcurrant juice. However, some interaction might occur when processing a multi-component mixture and results might vary depending on molecular weight, hydrophobicity, hydrophilicity and lipophilicity of the phenolic compounds.

#### 3.3.2. Effect of temperature

**Figure 5.**
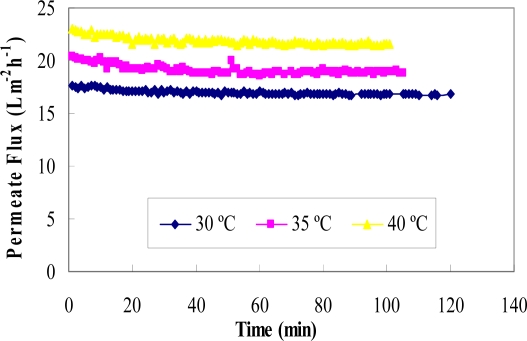
Permeate flux versus time for the binary mixture of rutin and glucose at three temperatures (T = 30 °C, 35 °C and 40 °C) and at TMP 4.

Figure 5 shows the effect of temperature on the permeate flux in ultrafiltration of glucose and rutin solutions. The temperature of the feed solution was varied while transmembrane pressure, pH, flow rate, rutin concentration and glucose concentration were kept constant at 35 °C, 5, 1 L/min, 0.25 g/L and 55 g/L, respectively.

In most membrane separation processes, permeate flux increases with increasing the feed solution temperature [35]. The results in this study, using a regenerated cellulose membrane, showed agreement with these previous studies. The effect of temperature on permeate flux may be attributed to the effect of temperature on the viscosity of the solution [29]. According to Darcy’s Equation, the viscosity of permeate decreases with the increase in the solution temperature, resulting in an increase in fluid flowing through the membrane. In addition, the solubility of rutin is better at higher temperatures, and therefore more is passed through the membrane.

**Figure 6.**
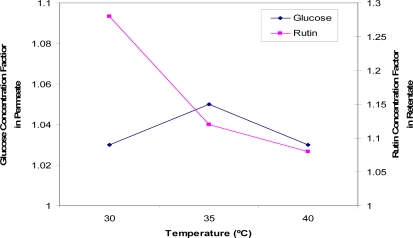
Concentration factors of glucose (in the permeate solution) and rutin (in the retentate solution) at three temperatures (T = 30 °C, 35 °C and 40 °C).

Figure 6 shows the effect of temperature on the concentration and separation of glucose (permeate side) and rutin (retentate side). The results suggest that the higher the temperature, the less rutin would be retained by the membrane, resulting in a smaller concentration enrichment being achieved in the retentate. Temperature had little effect on the permeation of glucose with only a slight variation observed. This may be due to a higher temperature, which favours the permeate flux by lowering fluid viscosity, and thus more rutin is transferred on the permeate side. The optimum temperature was found to be 30 °C, at which point a rutin concentration factor of 1.28 was achieved.

#### 3.3.3. Effect of pH

**Figure 7.**
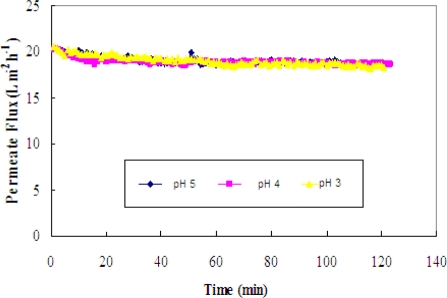
Permeate flux versus time for the binary mixture of rutin and glucose at three pHs (pH = 3, 4 and 5).

The effect of pH on the permeate flux is shown in Figure 7. The profiles suggest that the pH effects are not profound, as evident in the similar values of the permeate fluxes. Based on these flux values, any pH could be used for the separation.

**Figure 8.**
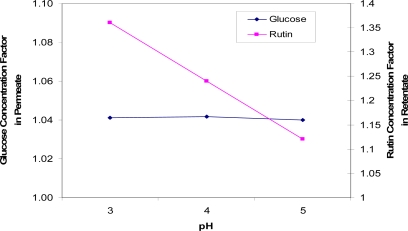
Concentration factors of glucose (in the permeate solution) and rutin (in the retentate solution) at three pHs (pH = 3, 4 and 5).

This conclusion is, however, not supported by data collected for the separation of rutin and glucose at various pH levels (Figure 8). The solution pH affected the membrane performance significantly, as the rutin concentration factor in the retentate increased by a factor of 1.2 when the pH of the feed solution decreased by 2 units. Therefore, the optimum pH to separate rutin and glucose is indicated to be approx. 3.

The optimum could have been different if the fouling had been considered in addition to the rutin concentration factor [36–38].

#### 3.3.4. Effect of feed rutin concentration

The permeate fluxes were compared at different feed rutin concentrations (0.1–0.5 g/L) with a constant glucose concentration of 55 g/L. It was observed that an increase in rutin concentration increased fouling and therefore resulted in some permeate flux decline.

**Figure 9.**
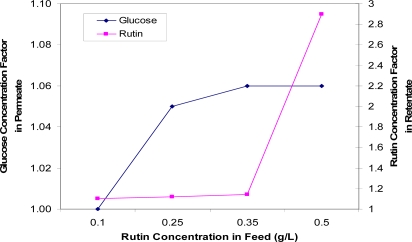
Concentration factors of glucose (in the permeate solution) and rutin (in the retentate solution) as a function of rutin concentration in the feed.

Figure 9 indicates that the more rutin initially in the feed, the more glucose recovered in the permeate solution and the more rutin retained in the retentate. This may be explained by there being a larger permeate flux drop at higher feed rutin concentrations, which indicates more mass being deposited on the membrane, resulting in more fouling. At a low feed rutin concentration of 0.1 g/L, both the glucose separated in the permeate solution and the rutin concentration in the retentate were low. By increasing the rutin concentration from 0.1 g/L to 0.5 g/L, rutin retention gradually increased and this was then followed by a dramatic increase between 0.35 and 0.5 g/L feed rutin concentration. Such a rapid increase may be due to the poor solubility of rutin at higher concentrations and possible aggregations. At high rutin concentrations, insoluble rutin may be more easily retained by the membrane and also form more fouling on the membrane.

#### 3.3.5. Effect of feed glucose concentration

**Figure 10.**
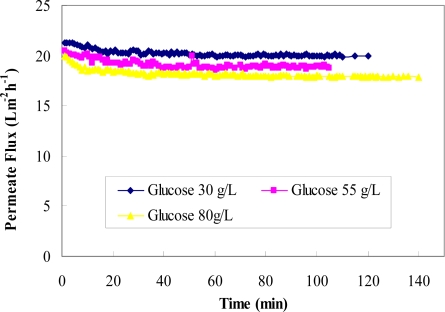
Permeate flux versus time for three glucose concentrations in the binary mixture (30 g/L, 55 g/L, and 80 g/L) at a constant rutin concentration of 0.25 g/L.

Figure 10 shows the permeate fluxes at varying feed glucose concentrations (30–80 g/L) with a constant feed rutin concentration of 0.25 g/L. These data suggest that an increase in glucose concentration decreased permeate flux. This phenomenon can be explained by the change in viscosity with the change in the feed glucose concentration.

**Figure 11.**
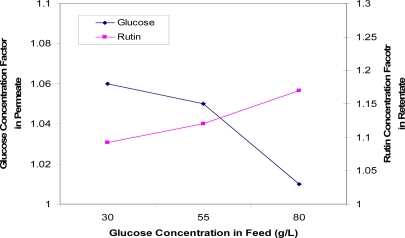
Concentration factors of glucose (in the permeate solution) and rutin (in the retentate solution) as a function of glucose concentration in the feed.

Figure 11 compares the concentration factors of rutin at various feed glucose concentrations. These data show that as glucose concentration increases, less glucose is being fractionated into the permeate solution and more rutin being retained by the membrane. Therefore, the optimum feed glucose concentration indicated is 55 g/L, at such concentration good separations of glucose and rutin are achieved on the respective sides of the membrane.

#### 3.3.6. Effect of feed flowrate

The permeate fluxes were found to be independent of the feed flow rate, as the concentration factors were only slightly changed (results not shown). The flow rates considered in the study were 1.0, 1.5 and 1.8 L/min and the other conditions were pH 3, TMP 4 and temperature 30 °C.

### 3.4. Diafiltration of Binary Feed

**Figure 12.**
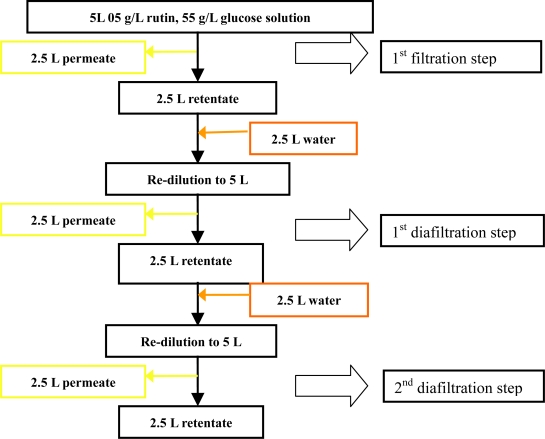
A schematic view of the steps involved in the diafiltration experiments.

Diafiltration of two components was performed under the following operating conditions: TMP 4 bar, temperature of 35 °C, pH 3, feed rutin concentration of 0.5 g/L, glucose concentration of 55 g/L and feed flow rate of 1 L/min. A simple diagram of the diafiltration of the mixture is shown in Figure 12. Starting with 5 L of fresh sample the ultrafiltration was continued until the retentate volume was reduced to half (2.5 L), then 2.5 L of deionized water was added to return the solution to the initial volume.

Observation of the permeate flux during the diafiltration process versus time demonstrated that the flux increased after each re-dilution step. This is because the removal of sugar in the permeate stream and addition of pure water led to an overall lowered sugar concentration in the re-diluted retentate and thus reduced osmotic pressure and increased flux [39].

After two diafiltration steps, 69% of rutin with a concentration factor of 1.36 was recovered and with only 11% of glucose in the retentate. The fouling deposits on the membrane were ca. 3.47 g.m^−2^ rutin, which is approx. 11.6% of the initial feed (Table 3).

### 3.5. Mass Balance

A mass balance for each experiment was carried out in order to obtain the amount of fouling deposits (as shown in Table 2) formed on the membrane. Since sugars do not form fouling, a mass balance with polyphenolics only was performed. The following assumptions were made:

- No leakage in the system
- Samples were an accurate representation of the actual situation.

The equation used to calculate the mass of deposits on the membrane, once the experiment had been completed, was (Equation 6):

(6) $$\text{M}=({\text{V}}_{\text{F}}\times {\text{C}}_{\text{F}}-{\text{V}}_{\text{R}}\times {\text{C}}_{\text{R}}-{\text{V}}_{\text{P}}\times {\text{C}}_{\text{P}})$$

where M is the mass of polyphenolics remaining on the membrane (g).

**Figure 13.**
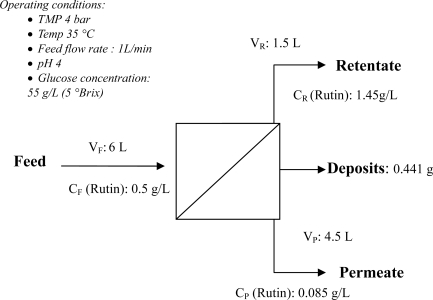
Mass balance for a mixture of rutin and glucose at the operating conditions mentioned in Figure 12.

The overall mass balance for the binary ultrafiltration sample using data obtained from run 1 (Table 2) is depicted in Figure 13. The mass balance shows that a total of 0.414 g of rutin from the feed stream (around 13.8% of the total rutin applied), was deposited on the membrane surface. The amount of fouling per unit area of membrane surface is summarized in Table 2.

## 4. Conclusions

The attempt in this investigation to separate and concentrate rutin from its two-component mixture with sugar, was successful. The following conclusions can be drawn in this study involving ultrafiltration through a commercially available membrane with MWCO 1000:

- Flux increases with increasing transmembrane pressure. However, after a certain pressure limit, permeate flux gradually levels off. An increase in transmembrane pressure increases the accumulation of polyphenolics and potentially causes the formation of fouling layers. The optimum transmembrane pressure can be suggested to be approx. 4 bar where the fouling is minimal.
- Flux increases with increasing feed solution temperature because of lower feed viscosity, and better solubility of rutin at higher temperatures.
- Separation and concentration of rutin are favored at lower temperatures (30 °C).
- Lowering pH can increase the retention of polyphenolics. The optimum pH was suggested to be approx. 3.
- Flux declines with the increase in concentration of rutin in the feed. However, the rutin concentration in the retentate increased simultaneously with that in the feed, suggesting that membrane fouling was severe and that the deposits formed prevented most of the rutin molecules from passing through the membrane pores.
- Higher cross flow rates can reduce membrane fouling by providing a shear force to sweep away deposited materials. At high feed flow rates, more polyphenolics were retained by the membrane with less sugar permeating through. The optimum feed flow rate was 1.5 L/min.
- The best performance of the process for the concentration of polyphenolics from sugar was judged to be when the rutin concentration factor and recovery in retentate were 2.9 and 72.5%, respectively; and the glucose concentration factor and recovery in permeate were 1.06 and 76.3% respectively.
- Diafiltration improved the membrane performance: it maintained the flux at higher levels and reduced the concentration of glucose in the retentate, with only approx. 11% of glucose being retained in the retentate after diafiltration.

## Figures and Tables

**Table 1.** Concentration factors for single component ultrafiltration.

	**Glucose**	**Sucrose**	**Rutin**	**Chlorogenic acid**
**Feed concentration(g/L)**	55	55	0.25	0.075
**Retentate concentration (g/L)**	57.8	60	0.33	0.077
**Concentration factor**	1.05	1.09	1.32	1.03

**Table 2.** Ultrafiltration of a binary mixture of glucose and rutin.

	**Process condition**				**Rutin content (g/L)**			**Glucose content(g/L)**			**Fouling deposits (gm^−2^)**

**Run**	**pH**	**TMP (bar)**	**Feed flowrate (L/min)**	**Temp (**°**C)**	**F**	**Ret**	**Per**	**F**	**Ret**	**Per**	

1	5	4	1	35	0.5	1.45	0.084	55	44.96	58.34	4.41
2	3	4	1	35	0.25	0.31	0.15	55	49.29	57.26	3.28
3	4	4	1	35	0.25	0.31	0.2	55	49.83	57.3	2.75
4	5	4	1	35	0.25	0.28	0.16	55	50.67	57.2	2.59
5	5	5	1	35	0.25	0.29	0.15	55	53.7	55.6	3.02
6	5	3	1	35	0.25	0.27	0.18	55	52.33	56.22	2.2
7	5	2	1	35	0.25	0.26	0.2	55	53.91	55.9	1.1
8	5	4	1	35	0.1	0.11	0.08	55	54.78	55.1	0.67
9	5	4	1	35	0.35	0.4	0.24	55	47.42	58.44	3.21
10	5	4	1	30	0.25	0.32	0.12	55	50.28	56.8	3.22
11	5	4	1	40	0.25	0.27	0.2	55	50.28	56.9	1.35
12	5	4	1	35	0.25	0.273	0.172	30	26.66	31.68	2.02
13	5	4	1	35	0.25	0.293	0.148	80	77.16	81.17	2.76
14	5	4	1.5	35	0.25	0.285	0.162	55	49.8	57.4	2.34
15	5	4	1.8	35	0.25	0.289	0.161	55	50.91	56.98	2.17

TMP = trans-membrane pressure, Temp = temperature, F = feed, Ret = retentate, Per = permeate.

**Table 3.** Concentration factor and recovery of glucose and rutin in retentate.

**Component**	**Concentration (g/L)**		**Concentration Factor (in retentate)**	**Recovery% (in retentate)**
	**Feed**	**Retentate**		

Rutin	0.50	0.682	1.36	69.00
Glucose	55.00	11.99	0.22	11.00
